# The Neuropeptide Neuroparsin-A Regulates the Establishment of Dominance Hierarchy in Bumblebees

**DOI:** 10.3390/ijms27010091

**Published:** 2025-12-21

**Authors:** Hao Wang, Yuwen Liu, Xiaohuan Mu, Wenjing Xu, Huiling Liu, Qiyao Yong, Xiaofei Wang, Yifan Zhai, Hao Zheng

**Affiliations:** 1 College of Food Science and Nutritional Engineering, China Agricultural University, Beijing 100083, China; wh691827@163.com (H.W.); 18813005256@126.com (Y.L.); miamu_00@163.com (X.M.); xuwenjing@cau.edu.cn (W.X.); liuhuiling2000@163.com (H.L.); yongqiyao1015@163.com (Q.Y.); xiaofei.wang@cau.edu.cn (X.W.); 2 Shandong Institute of Plant Protection, Shandong Academy of Agricultural Sciences, Jinan 250100, China; zyifan@tom.com

**Keywords:** *Bombus terrestris*, neuropeptide signaling, dominance hierarchy, social behavior, gene regulatory network

## Abstract

The regulation of reproductive division of labor in eusocial insects is pivotal for the evolution and maintenance of social organization. In *Bombus terrestris*, dominance hierarchies emerge among orphan workers through repeated agonistic interactions, forming distinct behavioral ranks. To explore the neural basis of this process, we combined behavioral assays with single-nucleus RNA sequencing to profile brain-wide gene expression across α-, β-, and γ-bumblebee workers. Our analyses revealed pronounced transcriptional divergence among Kenyon cells, which exhibited enrichment in synaptic, insulin, and MAPK signaling pathways. Among the neuropeptides examined, Neuroparsin-A was markedly upregulated in the Kenyon cells and glial cells of dominant workers, while its receptor, OR1, showed strong expression within Kenyon populations, suggesting a conserved neuropeptide–receptor axis in social Hymenoptera. Gene regulatory network inference further identified ecdysone-responsive transcription factors, including *br*, *Eip74EF*, *Hr38*, *Hr3* and *Hr4*, as key regulators linked to neural plasticity and behavioral differentiation. Together, our findings uncover a neuroendocrine mechanism in which Neuroparsin-A signaling coordinates brain transcriptional programs associated with dominance hierarchy formation in queenless bumblebee societies, offering new insights into the molecular underpinnings of eusocial behavior.

## 1. Introduction

Social behaviors are crucial for animals in locating mates, reproducing, and nurturing offspring, often requiring coordinated efforts among group members. Social insect colonies exemplify this phenomenon, consisting of overlapping generations that engage in shared brood care and demonstrate a clear division of labor between reproductive queens and non-reproductive workers [1]. In these societies, queens primarily handle reproduction, whereas workers perform all colony maintenance tasks, including brood care, nest defense, and foraging [2,3,4]. In honeybee colonies, callow workers initially perform nursing duties inside the hive before transitioning to foraging activities outside. In ants, smaller minor workers are typically responsible for foraging and brood care, while larger major workers function as soldiers defending the nest. The separation and subsequent specialization of these roles reflect the influence of Darwinian selection, promoting cooperation among colony members and enhancing the colony’s overall survival and reproductive fitness [3,5,6].

Bumblebees represent a key taxon for studying the phenomenon of eusociality, as they exhibit both cooperation and competition over reproduction, combining features of advanced eusocial species with traits characteristic of more primitive characteristics [7]. Although workers in bumblebee colonies perform distinct tasks, their division of labor is considerably more flexible than that of highly eusocial bees [8]. At the onset of the colony cycle, the queen monopolizes reproduction and suppresses ovarian activation in workers. However, as the colony’s life cycle progresses towards its end, social cohesion gradually deteriorates and transitions into a phase of reproductive competition and aggression. During this period, workers with activated ovaries concomitantly lay eggs and become aggressive, not only towards the queen but also towards other workers [9].

In contrast to the chaotic competition observed in queenright colonies, hierarchy formation in queenless conditions tends to be more stable, with reproductive hierarchies established through aggressive interactions and following a linear structure [10]. Queenless groups thus provide an ideal model to study reproductive competition among equally apt individuals, allowing the exploration of the mechanisms that govern hierarchy formation and reproduction among nestmates [11]. During the initial phase of establishing queenless groups, workers display a high level of agonistic behaviors until a clear dominance hierarchy is established [12]. In each group, the most dominant workers exhibiting increased overt aggression and threatening behaviors, such as pumping, buzzing, butting, and grappling, become reproductively active as the “α” worker They then possess the most developed ovaries compared to subordinate workers [12,13,14,15]. However, the neural mechanisms underlying these behaviors during the early stages of colony establishment remain unclear.

In social insects, physiology and behavioral processes are orchestrated by pleiotropic genetic networks in the brain, which integrates complex interactions among brain neuropeptides, hormones, and nutritional signals [16,17]. As key messenger molecules in the neuroendocrine system, neuropeptides play critical roles in coordinating physiological and behavioral processes across various animals by integrating internal and external cues from the environments [18]. In eusocial Hymenoptera, neuropeptides such as corazonin, and neuroparsin regulate caste-specific phenotypes, including foraging and oviposition [19,20,21,22,23]. In *Bombus terrestris*, the neuroendocrine system contributes to dominance rank regulation and task-related behavioral modifications [24,25]. Although recent genomic approaches have provided insights into the mechanisms mediating worker-worker conflict over reproduction in queenless *B. terrestris* worker groups [26], the molecular and neural mechanisms driving behavioral dominance, reproductive hierarchy, and the establishment of superorganismality remain to be elucidated. Furthermore, anatomical investigations reveal that the bumblebee brain possesses diverse and well-differentiated regions, including the optic lobes, ocellar synaptic plexi, mushroom bodies (MBs), central complex, anterior optic tubercle, and antennal lobes [27]. Emerging research utilizing single-cell RNA sequencing has uncovered a variety of cell populations in the bumblebee brain, exhibiting distinct gene expression profiles tied to social interactions among these insects [28,29]. Notably, behavior-specific neuro-genomic states are modulated by gene regulatory networks (GRNs), which outline the sophisticated connections between transcription factors (TFs) and their intended target genes, highlighting GRNs’ essential function in guiding gene expression adjustments [30]. Furthermore, GRNs connected to behavioral patterns appear in diverse brain regions that include assorted cell varieties, possibly displaying unique functionalities during changes in conduct [31]. Consequently, revealing the cellular variations in GRN architecture will yield significant understanding of the brain’s molecular traits related to different behavioral phases.

Here, we employed the single-nucleus RNA sequencing (snRNA-seq) in combination with the unique biology of *B.terrestris* to interrogate the neural correlates underlying the establishment of a dominance hierarchy in queenless groups. This study aims to characterize brain-wide transcriptional programs across different behavioral ranks, identify cell-type-specific neuropeptides and their potential receptors involved in regulating dominance-related behaviors, and explore gene regulatory networks linking neuroendocrine signaling to behavioral and reproductive differentiation.

## 2. Results

### 2.1. Bumblebee Workers Compete to Establish Dominance and Reproductive Hierarchies

**Figure 1:**
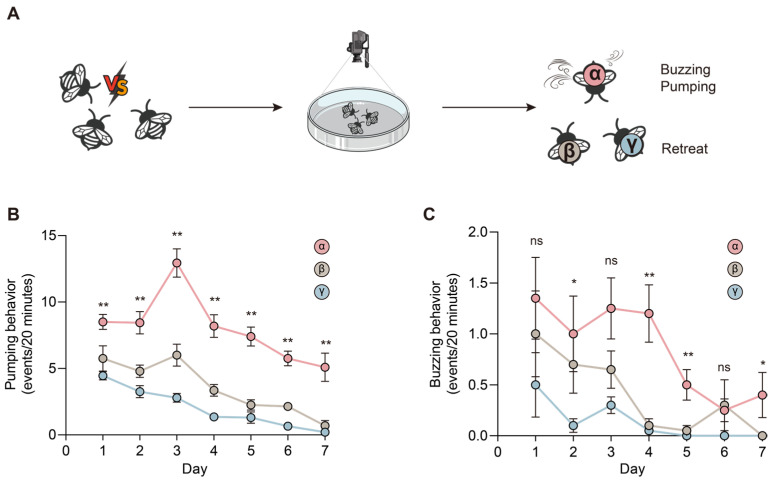
Dominance hierarchy in bumblebee workers is determined by agonistic interactions. (**A**) Schematic illustration of the dominance hierarchy establishment in queenless groups, each composed of three bumblebee workers. (**B**,**C**) Observed buzzing (**B**) and pumping (**C**) behavior events from different queenless groups (*n* = 10) during the establishment of dominance hierarchy. One-way ANOVA followed by Duncan’s test. Error bars represent mean SE. An asterisk (*) above the data point denotes statistical differences at *p*  <  0.05; a double asterisk (**) indicates *p*  <  0.01; ns, not significant.

To investigate the establishment of a dominance hierarchy, we built experimental groups consisting of three orphan worker bumblebees and monitored their agonistic interactions twice daily (9:00–9:20 and 21:00–21:20) for seven days (Figure 1A). We identified specific threatening behaviors between bumblebees, including “pumping” (characterized by a bee standing and making pumping movements with her abdomen; Video S1) and “buzzing” (a worker producing rapid, brief wing movements while facing another bee; Video S2) behaviors. By analyzing these behaviors, we found that “behaviorally dominant” bees exhibited significantly more pumping behavior from Day 1 and increased buzzing behavior from Day 2 onwards (Figure 1B,C, Tables S1 and S2). We further calculated the Dominance Index (DI) based on the retreat behavior [12] and found that the “behaviorally dominant” bee achieved an overwhelming advantage, winning 88% of encounters by Day 7 (Figure S1A, Table S3). Accordingly, the most dominant individual in the triplet was dubbed “α”, the median “β”, and the individual with the lowest rank “γ” [12]. We ensured that body size did not influence dominance by selecting bees with similar thoracic widths (~6.0 mm), confirming that dominance was behaviorally determined (Figure S1B). These findings demonstrate that α-worker bees, exhibiting the highest DI values and threatening behaviors, established dominance hierarchies within queenless group. Overall, bumblebees in these groups formed hierarchies through agonistic interactions.

### 2.2. KCs Are the Most Distinctly Regulated Cell Types in the Brains of Bumblebees Across Different Dominance Ranks

To investigate how brain gene expression differs across social ranks, we compared the bulk transcriptomes from the intact brains of α-, β-, and γ-worker bees. It only revealed a limited number of differentially expressed genes (DEGs) (Figure S2A,B), likely because cellular heterogeneity within the whole brain masked cell type–specific transcriptional differences associated with social rank. Therefore, we further dissected the brain heterogeneity using snRNA-seq between different ranks potentially masked in bulk profiling. We obtained 63,525 high-quality nuclei from the brains of α-, β-, and γ-worker bumblebees (Figure S3A–C). Additionally, over 85% of reads are mapped to the *B. terrestris* genome, with approximately 80% mapped to exonic regions, reflecting both data integrity and effective capture of protein-coding genes (Table S4). Together, these quality metrics confirm the reliability and reproducibility of our snRNA-seq datasets and provide a solid foundation for downstream analyses.

**Figure 2:**
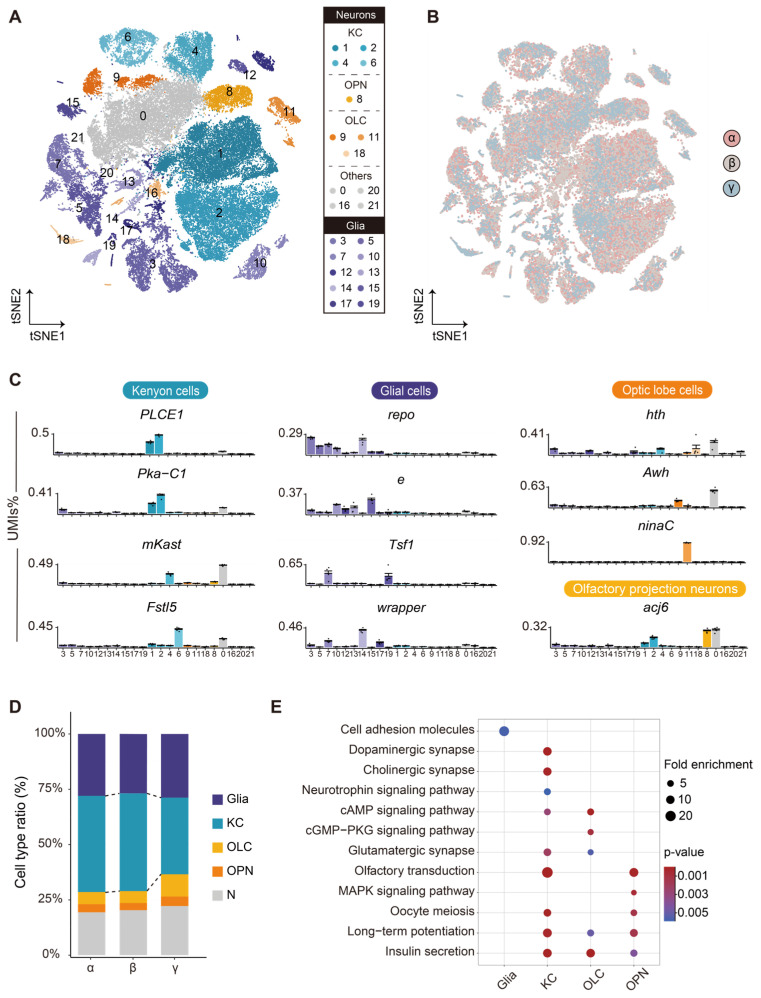
KCs are the most distinctly regulated cell types in the brains of bumblebees across different dominance ranks. (**A**) Annotated tSNE visualization of the clustering of 63,525 single-nucleus transcriptomes obtained from α-, β-, and γ-worker bumblebees (5 libraries). KC: Kenyon cells; OLC: optic lobe cells; OPN: olfactory projection neurons; Others: other neurons. (**B**) tSNE visualization of the clustering of brain cells from α-, β-, and γ-worker bees. (**C**) Selected marker genes for cell cluster annotation presented as the average percentage of total UMIs per cell per cluster. (**D**) Bar plots showing the proportion of different cell types in the brains of α-, β-, and γ-worker bees. (**E**) Representative KEGG pathways enriched (*p*-value < 0.05) by the DEGs upregulated in each cell type of α-worker bees relative to those of γ-worker bees. Dot sizes represent the fold enrichment, and dot colors indicate the *p*-value.

At a resolution of 0.2, we obtained 22 cell clusters (Figure 2A). Major cell types, including Kenyon cells (KCs), glia, optic lobe cells (OLCs), and olfactory projection neurons (OPNs), were annotated using known markers from single-cell transcriptomes of *Apis mellifera*, *Drosophila melanogaster*, and *Harpegnathos saltator* (Figure 2C) [32,33,34]. The global visualization using t-Distributed Stochastic Neighbor Embedding (tSNE) showed the consistency of cell types among the three ranks of bees (Figure 2B). However, there are variations in the relative proportions of cell types from α-, β-, and γ-worker bumblebees, with a notably higher percentage of KCs in α-worker bees (43.48%) compared to γ-worker bees (34.63%) (Figure 2D). Notably, the KCs of bumblebee brains exhibit a distinctive enrichment in synaptic, mitogen-activated protein kinase (MAPK), and insulin signaling pathways, which may regulate reproduction and food-seeking behaviors (Figure S4A) [16,35].

We identified the DEGs (|log_2_ Fold Change| > 0.5, FDR < 0.05) between bees of different ranks (Figures S4B and S5). Interestingly, we observed upregulation of *Vg* (*LOC100650436*) and *Y-f* (*LOC100651172*) genes across all major cell types in α-workers. In contrast, *Lethal (2) Essential for Life* (*l(2)efl*, *LOC100652068*) was downregulated in glia, KCs, and OLCs in α-worker bees. The KEGG analysis showed that numerous pathways, including the dopaminergic and cholinergic synapse, were uniquely enriched in the KCs of α-worker bees (Figure 2E). Importantly, key genes involved in these pathways showed elevated expression, including *calmodulin* (*LOC100649704*) and *Calcium/calmodulin-dependent protein kinase II* (*LOC100648089*, *CaMKII*), which are central to Ca^2+^-dependent synaptic plasticity, highlighting enhanced signal transduction capacity and inhibitory modulation in dominant individuals. These findings imply that KCs are the most distinctly regulated cell types in bumblebee brains across different dominance ranks, suggesting their crucial role in establishing dominance hierarchies within queenless groups.

### 2.3. Comparative Analyses of Neuropeptide Expression and Receptor Evolution in α- and γ-Worker Bee Brain Cell Types

Neuropeptides, among the most diverse signaling molecules in the insect nervous system, critically regulate behavioral plasticity and division of labor in social insects, with such as short neuropeptide F (sNPF), tachykinin-related peptides, and Neuroparsin-A playing key roles in modulating foraging behavior, aggression, and reproductive status [18,23].We analyzed caste-biased neuropeptides in *B. terrestris* (|log_2_ fold change| > 0.5, FDR < 0.05). A total of 14 neuropeptides that are involved in social insect behavior, neuroendocrine regulation, and caste differentiation were examined (Table S5) [23,36].

**Figure 3:**
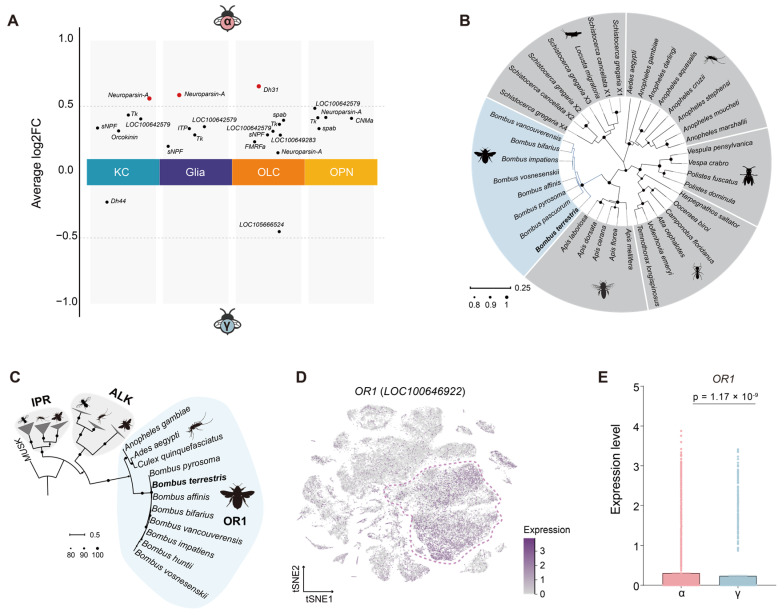
Comparative analyses of neuropeptide expression and receptor evolution in α- and γ-worker bee brain cell types. (**A**) Volcano plots showing insect neuropeptides in each cell type between α- and γ-worker bees (|Log_2_ Fold Change | > 0.5, FDR  <  0.05). (**B**) Neighbor-joining tree based on the amino acid sequences of *Neuroparsin-A* from bumblebees, honeybees, wasps, ants, mosquitos, and locusts. Node size indicates bootstrap values (1000 replicates). (**C**) Neighbor-joining tree of the RTKs from the orphan R1, IPR, and ALK clades of *Bombus*, *Drosophila*, and mosquitoes. The tree was rooted using the muscle-specific kinase from *D. melanogaster*. Node size indicates the bootstrap values (1000 replicates). (**D**) tSNE plot illustrating the expression level of *OR1* (*LOC100646922*) across all clusters. (**E**) Box plot showing *OR1* expression levels in KCs of α- and γ-worker bees. *p* values represent adjusted *p* values from Seurat’s FindMarkers function comparing expression in KCs of α-worker bees to that of γ-worker bees.

Our analysis revealed that *Neuroparsin-A* (*LOC100647295*) is significantly upregulated in KCs and glial cells within the brains of α-worker bumblebees (Figure 3A). Neuroparsin-A is a cysteine-rich neuropeptide that regulates insects’ gonadal development, including ants, mosquitoes, and locusts [37,38,39,40]. Phylogenetic analysis revealed the evolutionary conservation of Neuroparsin-A across eusocial Hymenoptera, forming species-specific clades for bumblebees and honeybees (Figure 3B and Figure S6). The genes from social bees shared the most remarkable similarity with those from ants and wasps.

Neuropeptides exert their physiological effects by binding to specific receptors, which in insects are predominantly classified into three major classes: G protein-coupled receptors, protein receptor kinases (PKRs), and receptor guanylyl cyclases [41]. Neuroparsin-A specifically interacts with the orphan R1 (OR1) clade within the receptor tyrosine kinase (RTK) subfamily, thereby regulating downstream signaling cascades [39,42]. Our analysis revealed that RTKs in bumblebees comprise multiple subfamilies, including anaplastic lymphoma kinase (ALK), the insulin-like peptide receptor (IPR), and OR1. OR1 homologs are widely distributed across various *Bombus* species, and *Bombus* OR1 clusters into an evolutionarily conserved branch sharing approximately 57% sequence similarity with mosquito orthologs (Figure 3C and Figure S7). The conservation of OR1 ligand-binding domains across *Bombus* species suggests a conserved neuropeptide-receptor signaling axis, potentially governing reproductive polyphenism. In *B. terrestris*, the *OR1* gene (*LOC100646922*) is highly expressed in KCs (Figure 3D) and shows markedly elevated expression in α-workers (Figure 3E), underscoring its likely involvement in the neuroendocrine regulation of caste-specific brain function.

**Figure 4:**
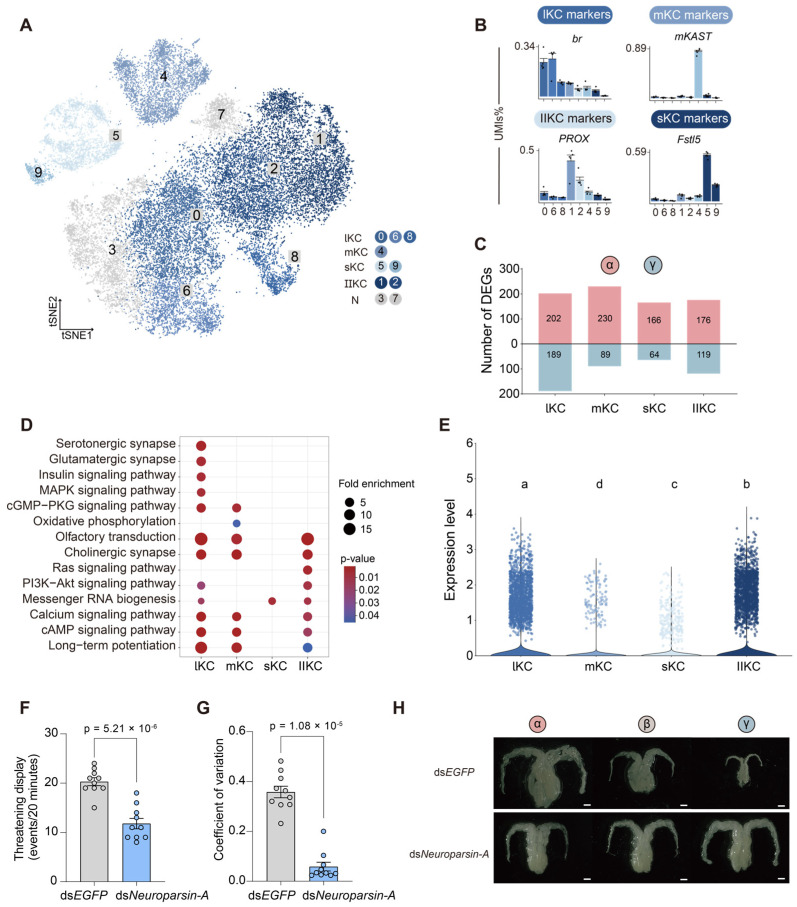
Subclustering and transcriptional characteristics of Kenyon cells in bumblebee brains. (**A**) tSNE visualization for the subclustering of Kenyon cells. sKC: Class-I small-type Kenyon cells; mKC: Class-I middle-type Kenyon cells; lKC: Class-I large-type Kenyon cells; IIKC: Class-II Kenyon cells. (**B**) Marker genes for the subclusters are annotated in KCs. Error bars represent mean SE. (**C**) Number of DEGs identified from different KC subtypes of α- and γ-worker bumblebees. (**D**) Representative KEGG pathways enriched (*p*-value < 0.05) by the DEGs upregulated in each KC subtype of α-worker bees relative to those of γ-worker bumblebees. Dot sizes represent the Fold enrichment, and dot colors indicate the *p*-value. (**E**) Violin plot of *OR1* expression across cell types. Statistical comparisons were performed using the Kruskal–Wallis test, and with a significance level of 0.05, adjusted by Benjamini–Hochberg correction. Different letters indicate statistically significant differences between groups. (**F**) Threatening displays of workers treated with *Neuroparsin-A* or *EGFP* dsRNA. Each dot represents the sum of threatening displays by three workers from the same queenless group (n = 10). Statistical analysis was performed using Student’s *t*-test. Error bars represent mean SE. (**G**) Relative variability in the length of ovarian terminal oocytes from the three workers within the same queenless group (n = 10), calculated as the coefficient of variation (ratio of SD to the mean). Statistical analysis was analyzed using the Mann–Whitney *U* test (n = 10). Error bars represent mean SE. (**H**) Representative ovaries of *Neuroparsin-A* and *EGFP* dsRNA groups 7 days after treatment. Scale bars = 1 mm.

To determine the expression pattern of the Neuroparsin-A receptor in KCs, we performed higher-resolution re-clustering of the KC population. At a resolution of 0.35, this analysis delineated 10 transcriptionally distinct subclusters that were annotated as large-type (lKCs), middle-type (mKCs), small-type (sKCs), and Class-II KCs (IIKCs) based on the expression of established subtype-specific marker genes (Figure 4A,B) [43,44,45]. DEGs in lKCs are associated with synaptic (serotonergic and glutamatergic), insulin, and MAPK signaling pathways, whereas DEGs in IIKCs are involved in the Ras signaling pathway, which regulates cell proliferation and differentiation regulation (Figure 4D).

Among KCs subtypes, lKCs are the most transcriptionally divergent subpopulation between α- and γ-worker castes (Figure 4C). Notably, the Neuroparsin-A receptor *OR1* showed strong α-worker-specific upregulation selectively in the lKC subclusters (Figure 4E). This specific expression pattern indicates that Neuroparsin-A signaling may primarily act through the binding of OR1 in lKCs of dominant α-worker bumblebees. This interaction likely plays a role in mediating caste-specific differentiation.

To further validate the importance of *Neuroparsin-A* in regulating bumblebee worker hierarchies, we knocked down its expression using a nanoparticle-mediated dsRNA delivery system. By feeding the nanoparticle-mediated dsRNA to bumblebees, the mRNA transcript level of the *Neuroparsin-A* gene in the brain was reduced by ~40% (Figure S8A). We found that ds*Neuroparsin-A* bumblebees showed fewer threatening displays 24 h after the treatment of *Neuroparsin-A* dsRNA (Figure 4F). Additionally, ds*Neuroparsin-A* bees from each queenless group had unvaried terminal oocyte lengths (Figure 4G,H), which were lower than those of α-worker bumblebees from the ds*EGFP* groups (Figure S8B). Taken together, these results indicate that Neuroparsin-A is a key neuropeptide that modulates both agonistic behavior and ovarian development, potentially serving as a molecular mediator in establishing dominance hierarchies among bumblebee workers. These results suggest that Neuroparsin-A acts as a systemic signal that selectively modulates higher-order neural circuits in dominant workers to reinforce reproductive dominance and behavioral plasticity.

### 2.4. Transcriptional Regulon Dynamics and Neuroparsin-A Regulation Associated with Behavioral Differentiation in Bumblebee Workers

Neuroparsin-A likely binds to its receptor to activate downstream signaling cascades, thereby modulating transcriptional programs within the brain through a complex gene regulatory network (GRN) which is a key driver of behavioral plasticity [23,39,46]. To further explore this regulatory landscape, we constructed a SCENIC database for the brains of *B. terrestris* and inferred GRNs by integrating co-expression and motif enrichment analyses.

**Figure 5:**
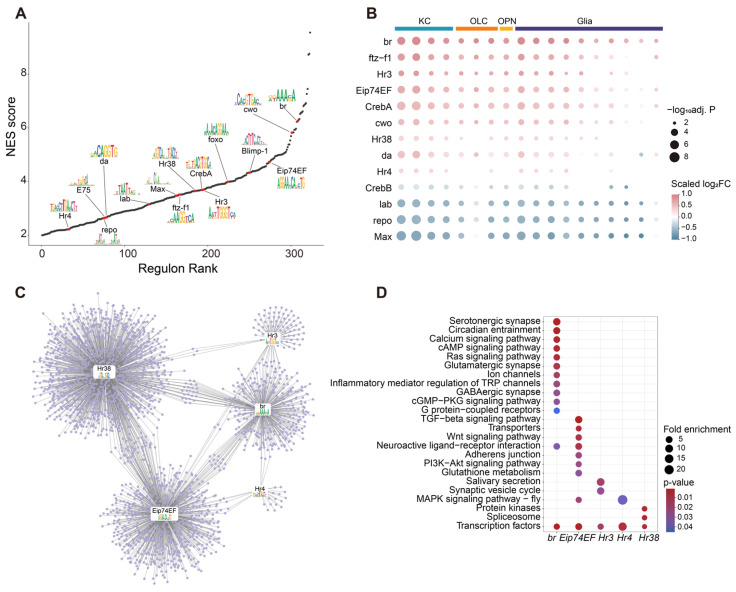
Transcriptional regulon dynamics and *Neuroparsin-A* regulation associated with behavioral differentiation in bumblebee workers. (**A**) Normalized expression scores (NES) and the size distribution of 323 regulons identified in bumblebee brains. Fifteen regulons of TFs related to behavioral differentiation and the ecdysone response are shown in red, along with their top-ranked motifs. (**B**) Differential regulon activity between α- and γ-worker bumblebee brains across cell types. Thirteen regulons previously associated with behavioral differentiation, and the ecdysone response are shown here. The size of the square represents the −log10(adj. P) derived from Seurat’s FindMarkers comparison between clusters of α-worker and γ-worker bees. The color scale indicates the normalized log_2_FC across different cell types. Log_2_FC  >  0 indicates upregulation in α-worker bees. (**C**) Regulatory network for the co-regulatory factors *br*, *Eip74EF*, *Hr38*, *Hr3*, *Hr4*. The target genes are shown in circles; the TFs and the corresponding motifs are shown in rounded rectangle nodes. (**D**) Representative KEGG pathways enriched (*p*-value < 0.05) by the DEGs upregulated in the target genes of the five TFs. Dot sizes represent the Fold enrichment, and dot colors indicate the *p*-value.

In total, 323 regulons were identified with significantly enriched motifs (NES score > 2) and their corresponding transcription factors (TFs). Notably, 15 TFs were highlighted as potential key regulators associated with behavioral differentiation and the ecdysone response (Figure 5A) [23,30,46]. Based on the constructed SCENIC database, we compared the activity of the regulons from different cell populations between α- and γ-worker bumblebees (Figure 5B). The *br* regulon exhibited markedly higher activity in most cell types of α-worker bumblebees. In particular, the *Eip74EF*, *Hr38*, *Hr3* and *Hr4* regulons, representing the ecdysone-responsive transcriptional modules, were significantly enriched in the KCs of α-worker bumblebees, indicating that the 20-hydroxyecdysone (20E) transcriptional cascade is specifically activated within the KCs of dominant individuals. Analysis of the regulatory network revealed extensive overlap in target genes among the five major ecdysone-responsive regulons, *br*, *Eip74EF*, *Hr38*, *Hr3* and *Hr4*, which collectively regulated 2336 genes (Figure 5C, Table S6). Specifically, target genes of the *br* regulon were significantly enriched in synaptic signaling pathways; for example, *Gat-a* (*LOC100647210*), *5-HT2A* (*LOC100648327*), and *mGluR* (*LOC10650736*) (Figure 5D). In addition, target genes of the *Eip74EF* regulon were associated with the PI3K-Akt and MAPK signaling pathways, which play pivotal roles in neuronal growth, hormone-mediated signaling, and transcriptional regulation downstream of ecdysone (Figure 5D) [17].

Together, these findings suggest that the ecdysone-associated GRN, potentially influenced by Neuroparsin-A signaling, plays a central role in transcriptional reprogramming underlying caste-specific behavioral and neuroendocrine differentiation.

## 3. Discussion

In this study, we demonstrated that dominance hierarchies are established through agonistic interactions among workers in queenless bumblebee groups. Behaviorally dominant individuals rapidly exhibited more frequent pumping and buzzing behaviors, leading to the formation of stable α, β, and γ ranks independent of body size. Transcriptomic and single-nucleus analyses revealed that dominance status is associated with distinct brain molecular profiles, particularly within KCs. SCENIC-based gene regulatory network inference further identified a highly active ecdysone-responsive module (led by *br*, *Eip74EF*, *Hr38*, *Hr3*, and *Hr4* regulons) that is selectively enhanced in α-worker KCs and target genes are significantly enriched in synaptic signaling, PI3K-Akt, and MAPK pathways. Among these, *Neuroparsin-A* and its receptor *OR1* were markedly upregulated in α-workers, suggesting a neuroendocrine pathway that links neural signaling with behavioral and reproductive differentiation. Knockdown of *Neuroparsin-A* further confirmed its essential role in modulating aggression and ovarian activation, highlighting a conserved mechanism underlying social dominance in eusocial insects.

Neuropeptides serve as key molecular mediators that regulate the formation and maintenance of social behaviors across diverse species [18,20,23]. A striking example is Neuroparsin-A, a conserved cysteine-rich neuropeptide whose behavioral roles vary markedly across ant species and castes. For instance, in *Camponotus floridanus*, reduced Neuroparsin-A expression in major workers enhances brood care [23], whereas in *Harpegnathos venator*, its knockdown influences hunting or defensive behaviors [38]. Thus, despite high sequence conservation, Neuroparsin-A has acquired divergent behavioral functions in different ant lineages. Here, we show in the bumblebee *B. terrestris* that *Neuroparsin-A* exhibits high expression in the brains of dominant α-workers, with knockdown suppressing threatening behaviors in queenless groups, thereby highlighting a critical role in the expression and maintenance of dominance-related behaviors. These results, together with the contrasting roles observed in ants, demonstrate that Neuroparsin-A is an evolutionarily conserved neuropeptide that has been repeatedly co-opted to regulate distinct social and reproductive behaviors across eusocial Hymenoptera. Its functional versatility highlights the remarkable plasticity of ancient signaling molecules in generating behavioral diversity and division of labor in highly social insects.

Neuroparsin-A regulates vitellogenesis [47,48], suggesting a mechanistic link between yolk production and brood care behavior, which could be further specialized among eusocial insect species [23,49]. In *Aedes aegypti*, the neuroparsin-like ovary ecdysteroidogenic hormone (OEH) is released from the brain in response to an amino acid-rich blood meal. It stimulates the ovaries to secrete ecdysteroid hormones, a key step in the egg formation process [50]. This suggests that nutrient status can regulate the synthesis of brain neuropeptide. Unlike honeybees, whose major royal jelly proteins are considered key components influencing queen development [51], their importance in bumblebees has been questioned. No differences were observed in the food composition provided by workers to larvae destined to become gynes or workers [52]. However, feeding frequencies were rather variable across individuals. The queen-destined larvae of *B. terrestris* are fed more frequently than worker-destined larvae, indicating a greater nutrient intake of queen-destined larvae [9]. Thus, the expression of neuropeptides in the brain may be associated with the potential variation in the nutrient level, such as amino acids [50], leading to behavioral difference.

Behavioral development in social insects is regulated by gene expression in the brain [34]. However, a recent study found that no genes in the brain showed significant differences between α- and γ-worker bumblebees [22]. This may be due to the limitations of bulk RNA-seq data. Our snRNA-seq analysis revealed variations in the proportions of cell types across different dominance ranks, with notable differences in KCs. KCs are the primary intrinsic neurons found in the insect mushroom body [53] and play a crucial role in sensory integration and memory functions [54]. In *A. mellifera*, the expression of immediate early genes in lKCs has been associated with aggressive behaviors of guards and nurses [55], suggesting that lKCs may play a role in behaviors related to caste differentiation. Furthermore, single-cell RNA sequencing analysis demonstrated that the expression of multiple key genes in different cell types of the ant brain is regulated in a stage-specific manner [56]. Bumblebees in the queenless groups established a dominance hierarchy within seven days. However, the most intense agonistic interactions occurred during the first three days (Figure 1B,C). Because our analyses focused on Day 7, the present study primarily captures neurogenomic and physiological states associated with established dominance rather than the initial formation of the hierarchy. Future research using time-series single-cell sequencing across early and late phases of group formation would provide deeper insight into the dynamic brain processes underlying agonistic interactions and dominance regulation in bumblebees.

Neuropeptides exert their effects on target cells primarily through interactions with specific membrane receptors [57]. Neuroparsins bind to PKRs, one of the three major types of membrane receptors that interact with peptide hormones [41]. In dipterans, PKRs are classified into two major families: the RTKs and receptor serine-threonine kinases, the latter being related to mammalian transforming growth factor beta receptors [41]. In *A. aegypti*, the OEH receptor is a member of the RTK subfamily, specifically within the OR1 clade, absent from *D. melanogaster*, lacking the neuroparsins [41,58]. Our phylogenetic analysis reveals that bumblebee species possess orthologs within the OR1 clade, showing similarity to the OEH receptor in mosquitoes (Figure 3C). This implies that neuroparsins may have conserved signaling functions across insects. However, functional divergence is evident in specific taxa. In social insects such as ants and bumblebees, neuroparsins modulate worker behaviors including aggression, dominance, and brood care, whereas in orthopterans like *Schistocerca gregaria*, they function as antigonadotropins [48]. Such divergence likely reflects lineage-specific adaptations of the neuroparsin-receptor signaling axis in coordinating behavior with social organization.

GRN regulating the changes in gene expression plays a crucial role in driving behavioral plasticity [46]. Our findings demonstrate that the activation of ecdysone-responsive regulons, such as those involving *br*, *Eip74EF*, *Hr38*, *Hr3*, and *Hr4*, in the KCs of α-worker bumblebees suggests a spatially targeted mechanism for enhancing neural plasticity and reproductive behaviors. This is evidenced by the higher regulon activity in α-workers, which correlates with enriched synaptic signaling pathways through target genes that are implicated in neurotransmitter modulation and metabotropic responses, potentially facilitating the aggressive and egg-laying tendencies observed in late-stage colonies [9]. In contrast, γ-worker bumblebees may maintain subordinate roles, prioritizing tasks like foraging and brood care to sustain early colony cohesion. Previous studies have shown that suppression of Neuroparsin-A signaling is associated with transcriptional remodeling involving neuronal, developmental, and ecdysone-responsive genes, including *Hr38*, *Blimp-1*, and *usp*, suggesting that Neuroparsin-A may modulate endocrine-linked transcriptional networks [23]. Thus, Neuroparsin-A could serve as a molecular link that integrates hormonal signals with behavioral outcomes in *B. terrestris* workers.

When deprived of a queen, worker bees engage in agonistic behaviors, reprograming brain gene networks and initiating a cascade of neuropeptide-mediated signaling. Our findings highlight Neuroparsin-A as a central neuropeptide regulating dominance-related behaviors in bumblebees, thereby contributing to the establishment of worker hierarchies following queen loss. Together with evidence from ants and other insects, these results suggest that the neuroparsin-receptor signaling axis constitutes a conserved yet behaviorally versatile mechanism, co-opted in different lineages to regulate context-specific social behaviors ranging from aggression to brood care. Future studies integrating time-resolved single-cell transcriptomics with experimental manipulation of neuropeptide signaling will be crucial to uncover how conserved molecules like Neuroparsin-A are diversified to meet the ecological and social demands of eusocial insects.

## 4. Materials and Methods

### 4.1. Bumblebees

*B. terrestris* colonies were obtained from the Institute of Plant Protection, Shandong Academy of Agricultural Sciences (Jinan, China). We removed all the emerged adult workers from the colonies and housed each colony in a nesting box (16 × 11 × 4.5 cm) placed in an environmental chamber (29 °C; 55% ± 5% RH, in the dark). Then we collected newly emerged callow workers up to about 12 h of age who do not yet show yellow pigmentation. We measured the thoracic width of all the callow workers as an index for body size and only kept those with a thoracic width of 6.0 mm ± 0.5 mm. We established queenless groups with three orphan workers. Each bee was labeled with a colored tag (red, yellow, blue) and placed in a plastic Petri dish (9 × 1.5 cm). Bumblebees were fed on sterilized pollen and sugar syrup (50% sucrose solution, *w*/*v*) ad libitum.

### 4.2. Behavioral Observations

The behaviors of the callow bees from each queenless group were recorded (Basler acA1300-60gmNIR, Ahrensburg, Germany) twice a day (9:00–9:20, 21:00–21:20) during days 1–7. The recorded videos were digitized and analyzed using the EthoVision XT 17 software (Noldus, Wageningen, The Netherlands). During the establishment of the queenless group, the orphan workers typically show agonistic behaviors and create clear dominance hierarchies [12]. We documented threatening displays for each bee, including “buzzing,” where a worker produces rapid, short wing vibrations while facing another bee, and “pumping”, which involves a bee standing and facing a nestmate while performing distinct dorsoventral pumping movements with her abdomen (Videos S1 and S2).

We calculated each bee’s DI to estimate the dominance of individual bumblebees [12]. Briefly, the DI represents the proportion of encounters between each pair of bees where the focal bee did not retract from all encounters.

$$DI=1-\frac{Retractions}{Total\;encounters}$$

In each group, we designated the worker with the highest DI value as “alpha” (α), followed by “beta” (β), and “gamma” (γ) in descending order of DI.

### 4.3. Tissue Collection

After completing the behavioral observations on Day 7, the brains from individual α-, β-, and γ-worker bumblebees were collected, following the dissection procedures illustrated in Figure S9. Bumblebees were fixed on beeswax using two insect needles through the thorax. After removing the head cuticle, dissected brains were placed on a microscope slide and immersed in RNAlater (Thermo Fisher Scientific, Waltham, MA, USA). The salivary glands, hypopharyngeal glands, three simple eyes, and two compound eyes were removed. The intact brain tissue was transferred into a 1.5 mL sterile centrifuge tube, quickly stored in liquid nitrogen, and then transferred to −80 °C for sequencing analysis.

Microscopic scissors were used to cut small incisions through the lateral and ventral sides of the abdomen, and the internal organs were then immersed in RNAlater, as shown in Figure S9. Ovaries were dissected. We gently transferred the ovaries into a drop of 1× PBS (Solarbio, Beijing, China) on a microscope slide. We used an ocular ruler to measure the length of the four largest terminal oocytes from both ovarioles. The mean length of the terminal oocytes was used as an index for the ovarian state [59]. Ovarian measurements and behavioral assays were performed on three workers from each of 10 queenless groups (n = 10 groups), providing biological replicates for all analyses.

### 4.4. RNA-seq of Bulk Brain Tissues

Total RNA was extracted from individual brains using the RNA-easy Isolation Reagent (Vazyme Biotech, Nanjing, China). RNA integrity was detected by agarose gel electrophoresis (agarose gel concentration: 1%, voltage: 150 V, electrophoresis time: 30 min), and the purity was checked with the NanoPhotometer™ P330 (IMPLEN, Westlake Village, CA, USA). For each worker type (α-, β-, and γ-worker), four independent RNA libraries were prepared from individual brains, providing biological replicates for each condition. Total brain RNA was used to construct the sequencing library using the NEBNext Ultra^TM^ II RNA Library Prep Kit for Illumina (New England BioLabs, Ipswich, MA, USA). The library was initially quantified by Qubit2.0 Fluorometer. The library preparations were then sequenced on an Illumina NovaSeq 6000 platform (Illumina, San Diego, CA, USA), and 150 bp paired-end reads were generated. The sequencing quality of each sample was assessed using FastQC v0.11.5 with default parameters. An index of the reference genome of *B. terrestris* (GCF_910591885.1_iyBomTerr1.2_genomic) was constructed using HISAT2 v2.0.5 [60], and the FastQC trimmed reads were aligned to the constructed index using HISAT2 v2.1.0 with default parameters. Gene expression was quantified using HTSeq v0.7.2 [61] with mode “union”. Only reads mapping unambiguously to a single gene are counted.

Differential gene expression analysis was performed using the DESeq2 v1.20.0 [62]. The resulting *p*-values were adjusted using Benjamini–Hochberg’s approach to control the false discovery rate. The thresholds for significant differential expression were FDR  <  0.05 and |Log_2_ Fold Change | > 1. Principal Coordinates Analysis (PCoA) was performed using vegan v2.6-4. The distance matrix was calculated using the Bray–Curtis method, and statistical significance was assessed using permutational multivariate analysis of variance (PERMANOVA).

### 4.5. Single-Nucleus Library Preparation and Sequencing

In brief, the frozen bumblebee brains (two brains for each library; five libraries in total for α-, β-, and γ-workers) were transferred into a 2 mL Dounce homogenizer containing 2 mL of ice-cold 1× Homogenization Buffer. This buffer consisted of 0.1 mM Tris-HCl (pH = 7.5, Thermo Fisher), 0.1 mM NaCl (Invitrogen, Carlsbad, CA, USA), 0.03 mM MgCl_2_ (Thermo Fisher), 0.1 mM DTT (Sigma, St. Louis, MO, USA), 1× Protease inhibitor cocktail (Roche, Basel, Switzerland), 0.4 U/μL RNase inhibitor (BGI, Beijing, China), and 0.1% NondietP-40 (Roche). After being placed with ice for 10 min, the tissues were homogenized until no visible tissue remained. The homogenate was filtered through a 30 μm strainer into a 15-mL tube and centrifuged at 500× *g* for 5 min at 4 °C to pellet the nuclei. The pellet was resuspended in PBS containing 0.2 U/μL RNase inhibitor and 1.5% bovine serum (Solarbio, Beijing, China) albumin. After another centrifugation step, the nuclei were resuspended in PBS containing 0.04% bovine serum albumin and 0.2 U/μL RNase inhibitor. The final cell concentration of single-nucleus suspension was adjusted to 1000 nuclei per μL for library preparation. The snRNA-seq libraries were constructed using the DNBelab C Series Single-Cell Library Prep Set (MGI, 1000021082) [63] and sequenced on the DIPSEQ-T1 platform at the China National GeneBank (Shenzhen, China) with read lengths of 41 bp for Read 1 and a 100 bp read length for Read 2. In total, five independent libraries were prepared from α-, β-, and γ-worker brains, yielding 63,525 single-nucleus transcriptomes (see Supplementary Table S4 for detailed library information).

### 4.6. snRNA-seq Data Analysis

Raw sequencing data from DNBSEQ-T1 were filtered and demultiplexed using PISA v0.2 [64]. The quality-controlled reads were then aligned to the reference genome of *B. terrestris* (GCF_910591885.1_iyBomTerr1.2_genomic) using STAR v2.5.1b [65]. The bam files were sorted using Sambamba v0.7.0 for downstream analysis [66]. Reads were annotated against the *B. terrestris* GTF file using “PISA anno”. The barcode rank plots were generated, and the ambient RNA noise was reduced based on the cutoff point between empty and nonempty barcodes in SoupX v1.4.8 [67]. The nucleus versus gene UMI count matrix was generated using PISA.

The Seurat v4.4.0 [68] pipeline was executed in each library for cell quality control. Cells were sorted based on the number of expressed genes (retaining those in the 5th to 95th percentile), with each gene required to be detected in at least three cells. Cells with high mitochondrial content (percentage of UMIs classified as mitochondrial genes < 0.03) were removed. The UMI count matrix was normalized using the “NormalizeData” function, and the most variable genes were extracted using the “FindVariableFeatures” function. Principle components (PCs) were calculated based on the variable genes using the “RunPCA” function.

DoubletFinder v2.0 [69] was used to remove potentially doublet cells. The expected doublet rate was estimated as 5%, with pseudo-doublets generated with pN (the number of artificial doublets) set to 0.25 and pK (the neighborhood size) set to 0.01. Cells were identified as doublets based on their rank order in the distribution of the proportion of artificial nearest neighbors (pANN), and cells with the top pANN values (>pK) were predicted as doublets. The “FindIntegrationAnchors” and “IntegrateData” functions were executed to integrate all libraries into a single dataset. Batch effects were corrected using the first 30 principal components (reduction = “rpca”, normalization method = “LogNormalize”).

### 4.7. Cell Type Annotation

To select an appropriate resolution for clustering snRNA-seq datasets, we first performed graph-based unsupervised clustering using the “FindNeighbors” and “FindClusters” functions with a series of resolutions. We then manually checked the visualization of the movement of cells between clustering branches using the R package clustree v0.5.0 [70] to determine the version for stable clustering. A clustering resolution 0.2 was chosen for the brain dataset. We annotated the brain cell types using the established marker genes well-studied in *A. mellifera*, *D. melanogaster*, and *H. saltator*. To define homologous genes between *B. terrestris* and these other insects, we used orthologs provided by OrthoDB v11 [71]. Also, we included the protein sequences with two-way reciprocal best hits identified by DIAMOND v0.9.25 (e-value < 1× 10^−5^, percentage of identical matches > 30%) [72].

### 4.8. SCENIC Analysis

We used motif databases tailored for cisTarget and SCENIC analyses in *B. terrestris*. TFs with motif modules from *A. mellifera* (CNP0003087) were collected, and 602 orthologous TFs corresponding to 9737 position weight matrix (PWM) models were identified in *B. terrestris* through orthology analysis. A cisTarget database was then generated using the “create_cisTarget_databases” pipeline (https://github.com/aertslab/create_cisTarget_databases, (accessed on 15 September 2025)). PWMs were evaluated across regulatory sequences spanning 5 kb upstream and 2 kb downstream of each gene, with the top-scoring motif-gene linkages forming the basis for gene-motif rankings [28].

We pinpointed regulons exhibiting differential activity among cell types by adhering to the SCENIC protocol [73], executed through pySCENIC v0.12.0 [74]. GRNBoost2 was used to deduce co-expression modules and quantify interaction strengths between TFs and their targets. Putative direct-binding targets were delineated via cisTarget, retaining only those motifs with normalized enrichment scores (NES) exceeding 2.0. Finally, AUCell was employed to assess regulon activity, evaluating the enrichment of target genes in each cell.

For each regulon, the log_2_ fold change (log_2_FC) between α- and γ-worker bees was calculated within each cluster at the single-cell level. To assess significance, differential regulon activity between α- and γ-worker bees across cell types was determined using the “FindMarkers” function in the Seurat package, implemented with the Wilcoxon rank-sum test.

### 4.9. DEGs and Enrichment Analysis

The “FindMarkers” function was used to identify DEGs between cell clusters based on the Wilcoxon rank-sum test (|log_2_ FoldChange| > 0.5 and FDR < 0.05). The Kyoto Encyclopedia of Genes and Genomes (KEGG) database was used to explore the biological pathways in which the DEGs were enriched. The KEGG pathway enrichment analysis was performed using clusterProfiler v4.6.2 [75].

### 4.10. Phylogenetic Analysis of Neuroparsin-A and the RTKs

We identified the *Neuroparsin-A* genes and the RTKs orthologs in different species of bumblebees, honeybees, wasps, ants, mosquitoes, and locusts through BLASTp 2.13.0 searches against the National Center for Biotechnology Information non-redundant (NCBI nr) database. Amino acid sequences were aligned using the Clustal W and Multiple Sequence Alignment by the Fast Fourier Transform program [76]. Neighbor-joining trees were constructed by MEGA 7.0 with 1000 bootstrap replicates [77].

### 4.11. RNAi for Neuroparsin-A and Quantitative PCR

To generate double-stranded RNA for the *Neuroparsin-A* gene, the coding regions were amplified from brain cDNA using forward and reverse primers that contained the T7 promoter sequence at their 5′ ends (Table S7). Amplification reactions were conducted in 50 μL solutions with 20 μL ddH_2_O, 25 μL 2 × Phanta Max Master Mix (Vazyme Biotech), 2 μL of forward primer, 2 μL of reverse primer, 1 μL of cDNA template. The PCR cycling conditions were as follows: 95 °C for 3 min, followed by 35 cycles of 95 °C for 15 s, 60 °C for 15 s, and 72 °C for 30 s, and a final extension step of 72 °C for 5 min. The partially amplified segments of the genes were cloned into the 5 min TA/Blunt-Zero Cloning Kit (Vazyme Biotech) and verified by Sanger Biotech sequencing. The dsRNA for RNAi was produced using the MEGAscript™ RNAi Kit (Thermo Fisher Scientific). The fragment amplified from the *EGFP* gene was used as a control [78]. The dsRNA was stored at −80 °C until it was needed. The sugar syrup with dsRNA was replaced daily for 7 days, and each bumblebee ingested about 5 μg of dsRNA per day. We performed daily behavioral observations on days 1 to 7. After 7 days, we collected the bumblebee brains to assess RNAi efficiency and measured the length of the terminal oocytes. RNA extraction and quantitative PCR.

Total RNA was extracted from individual brains and fat bodies using the RNA-easy Isolation Reagent (Vazyme Biotech). cDNA was synthesized from 1 μg RNA using the HiScript III RT SuperMix for qPCR (+gDNA wiper) (Vazyme Biotech). For gene expression analyses, qPCR was performed in six biological replicates with the gene-specific primers (Table S7) in QuantStudio 1 Real-Time PCR Instrument (Thermo Fisher Scientific) using ChamQ Universal SYBR qPCR Master Mix (Vazyme Biotech). The β-actin gene of *B. terrestris* was chosen as the control [28], and the relative expression was calculated using the 2^−∆∆CT^ method [79].

### 4.12. Statistical Analysis

Statistical analyses were conducted using R v4.4.1 and SPSS v26 [80,81]. The dynamics of DI, pumping, and buzzing event numbers were analyzed using one-way ANOVA followed by Duncan’s test. The arcsine square-root transformation (arcsin$\sqrt{p}$) was applied to DI prior to analysis. Thorax width was analyzed using one-way ANOVA followed by Duncan’s test after verifying data for normality using Kolmogorov–Smirnov test for normality. For threatening displays, we used either two-tailed Student’s *t*-test or the nonparametric Mann–Whitney *U* test, depending on whether the data followed a normal distribution.

## 5. Conclusions

In summary, our study uncovers a neuroendocrine mechanism underlying the formation of dominance hierarchies in queenless *B. terrestris* workers. We demonstrate that Neuroparsin-A signaling coordinates brain transcriptional programs, particularly in KCs and glial cells, to regulate behavioral differentiation and neural plasticity associated with social rank. Furthermore, our gene regulatory network analysis highlights ecdysone-responsive transcription factors as key modulators linking neuroendocrine signaling to dominance-related behaviors. Together, these findings provide novel insights into the molecular and cellular bases of eusocial behavior and advance our understanding of how neuropeptide signaling orchestrates reproductive division of labor in social insects.

## Data Availability

The raw data of the snRNA-seq have been deposited into the CNGB Sequence Archive of China National GeneBank DataBase with accession numbers CNP0006161. The accession numbers for the RNA sequencing data are PRJNA1147464.

## Supplementary Materials

The following supporting information can be downloaded at: https://www.mdpi.com/article/10.3390/ijms27010091/s1.
