# Supplementary material for: The Neuropeptide Neuroparsin-A Regulates the Establishment of Dominance Hierarchy in Bumblebees
Source: Int J Mol Sci. 2025 Dec 21;27(1):91. doi: 10.3390/ijms27010091 (PMC12785732; doi:10.3390/ijms27010091)
Supplement: Supplementary file 1 [file ijms-27-00091-s001.zip › Supplementary Figures & Videos.pdf]

Supplementary Figures

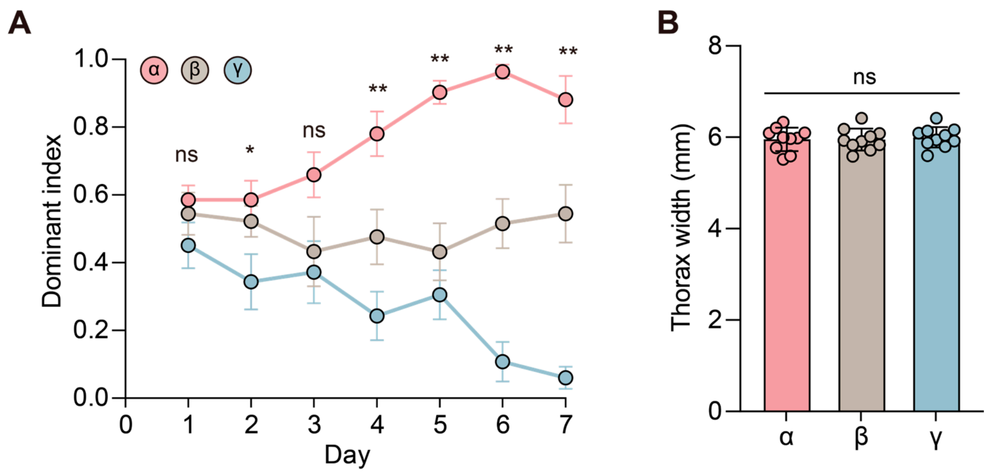

Figure S1. Dominance hierarchy and worker types in queenless bumblebee groups.

(A) The Dominance Index values were calculated for each bee individual within the same group (n = 10) based on their behaviors during all encounters. One-way ANOVA followed by Duncan's test. Error bars represent mean SE. An asterisk (*) above the data point denotes statistical differences at $p < 0.05$; a double asterisk (**) indicates $p < 0.01$. (B) The queenless groups are composed of α-, β-, and γ-worker bumblebees (from n = 10 queenless groups) with similar body sizes. One-way ANOVA followed by Duncan's test. Error bars represent mean SE.

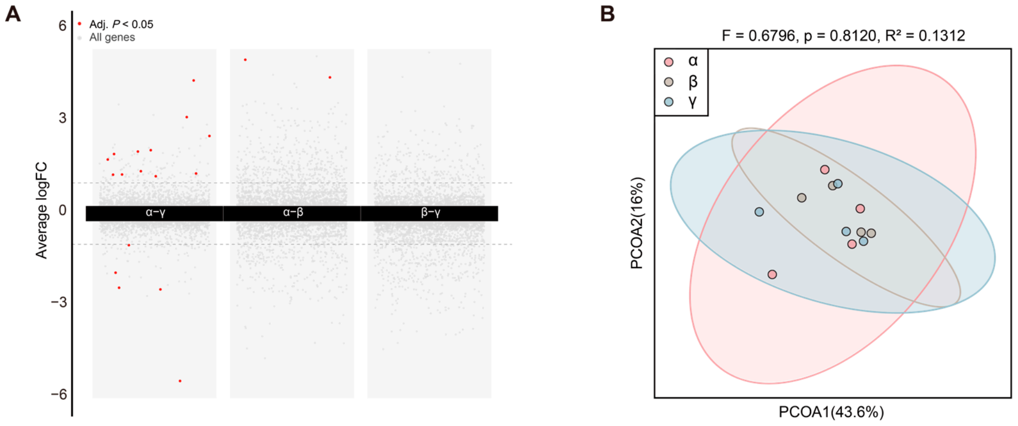

Figure S2. RNA-seq analysis from brains of α-, β-, and γ-worker bees.

(A) Volcano plots showing differentially expressed genes among α-, β-, and γ-worker bumblebees (|Log2 Fold Change | > 1, FDR < 0.05). (B) PCoA analysis based on average expression profile from brain transcriptome of α-, β-, and γ-worker bumblebees using the Bray-Curtis Dissimilarity metric. Ellipses were drawn using the standard deviation of points within the respective groups.

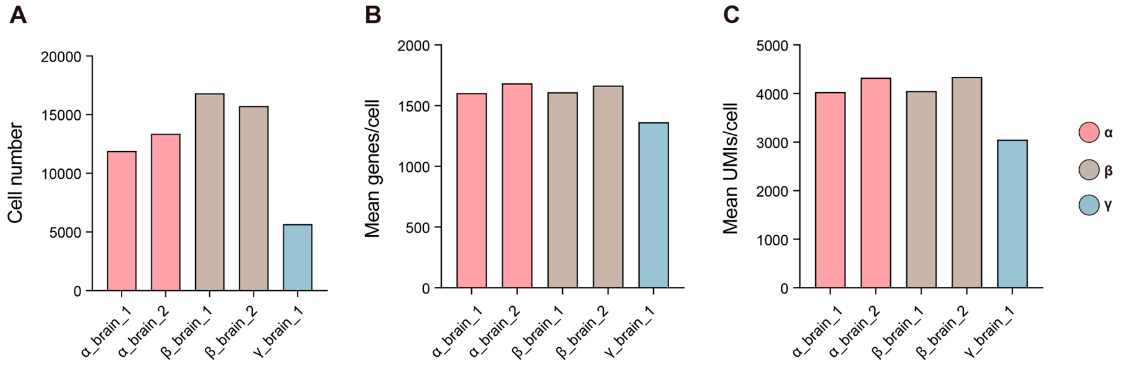

Figure S3. Quality control metrics for the single-nucleus RNA-seq datasets.

(A-C) Number of cells (A) and the average number of genes (B) and UMIs (C) detected in nuclei from each treatment across intestinal segment. UMI: Unique Molecular Identifier.

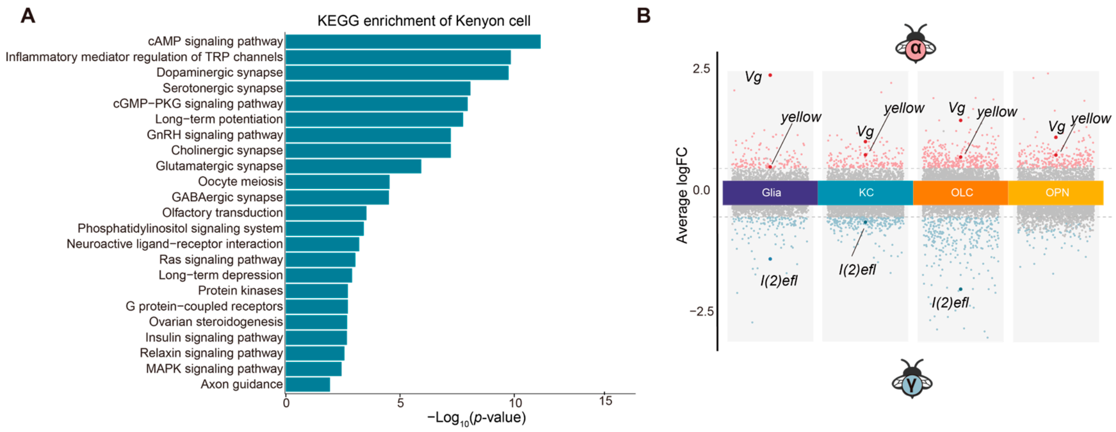

Figure S4. Single-nucleus transcriptomes from the brains of α-, β-, and γ-worker bees.

(A) The KEGG pathway is enriched by genes that are specifically expressed in Kenyon cells compared to other cell types. (B) Volcano plots showing differentially expressed genes in each cell type between α- and γ-worker bumblebees (|Log2 Fold Change | > 0.5, FDR < 0.05).

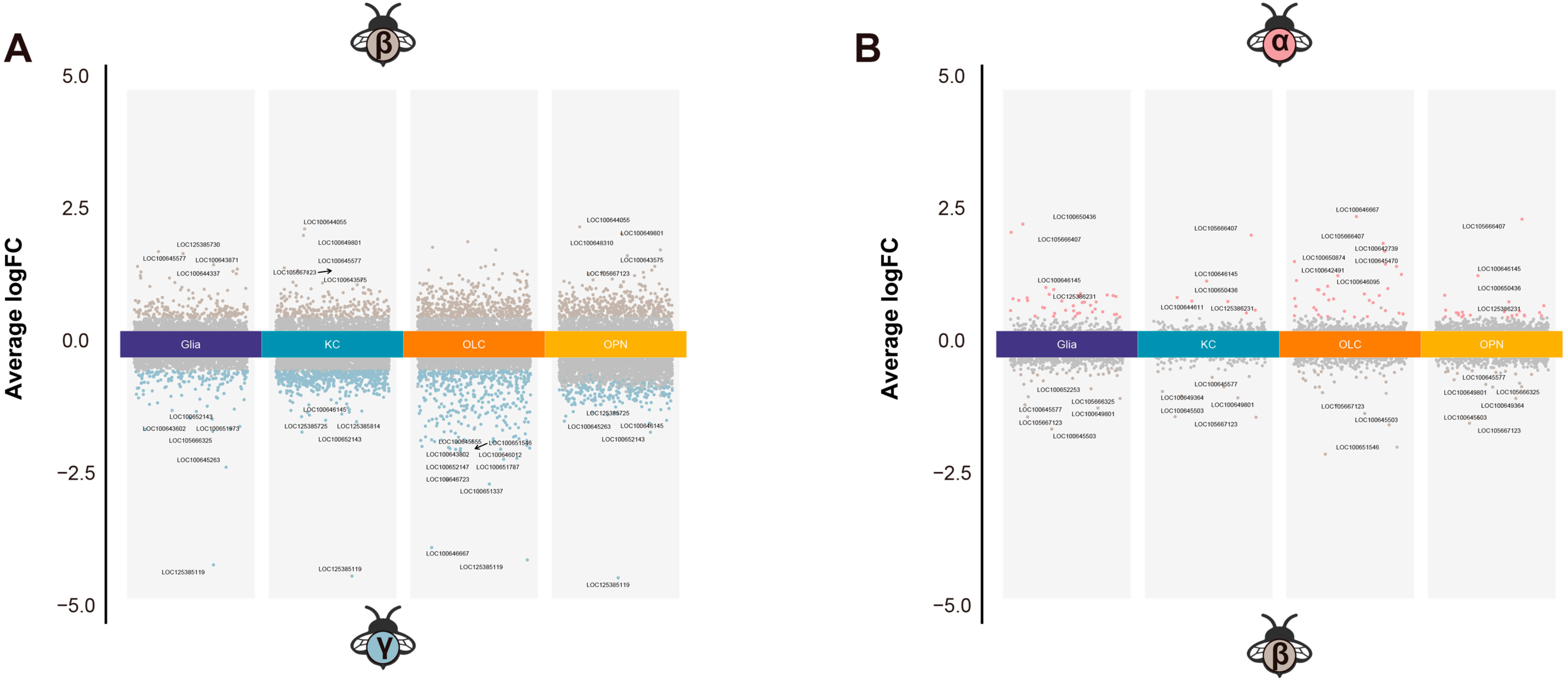

Figure S5. Analysis of differentially expressed genes among bees in the queenless groups.

Volcano plots showing differentially expressed genes across various cell types in comparisons between β- and γ-worker bumblebees (A) and between α- and β- worker bumblebees (B) (|Log2 Fold Change | > 0.5, FDR < 0.05).

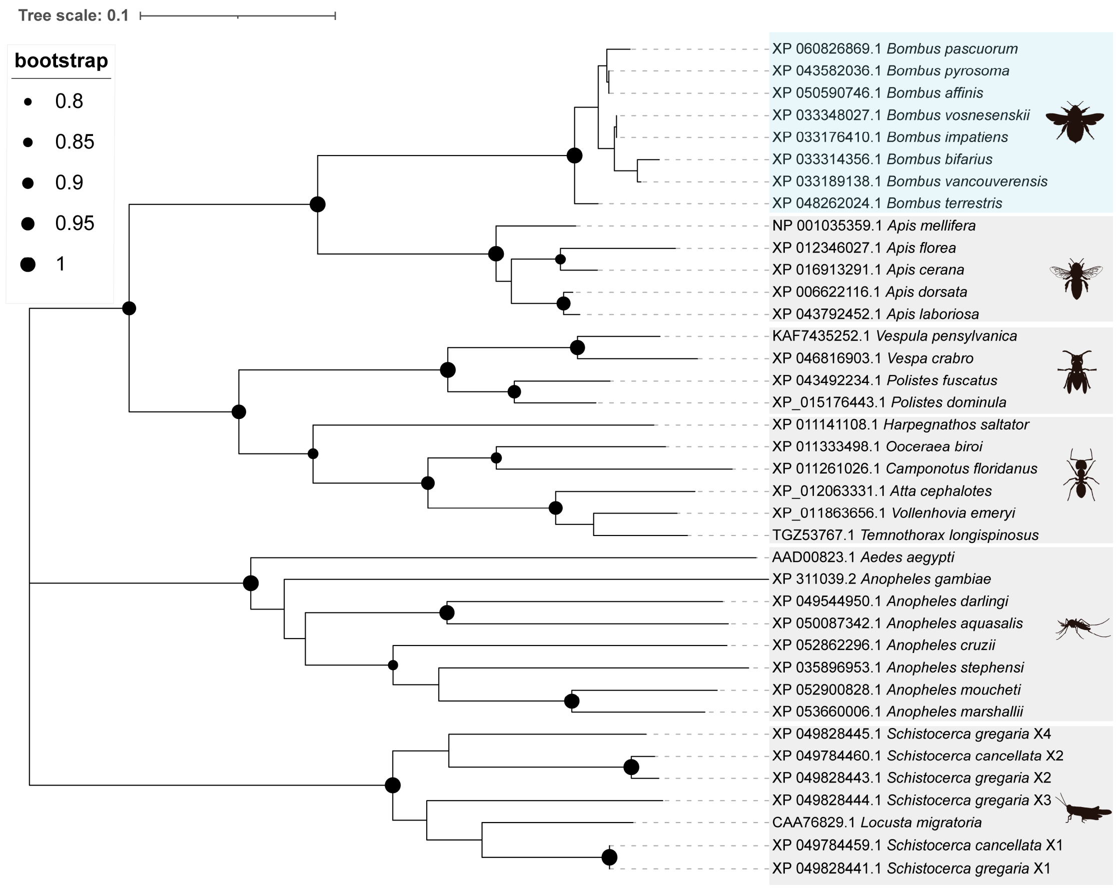

Figure S6. Neighbor-joining tree based on the amino acid sequences of *Neuropasrsin-A* from bumblebees, honeybees, wasps, ants, mosquitos, and locusts. Node size indicates bootstrap values (1,000 replicates). The accession numbers of amino acid sequences are labeled in front of the species names.

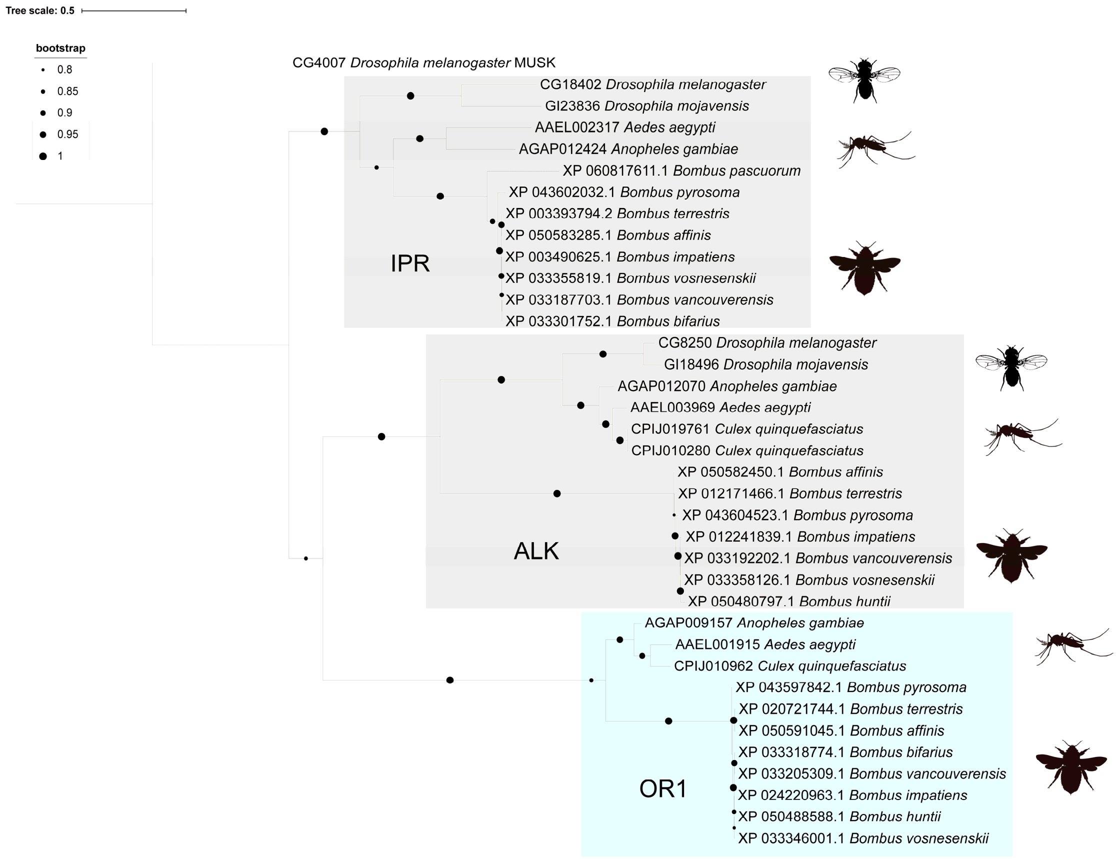

Figure S7. Neighbor-joining tree of the receptor tyrosine kinases from the orphan R1, Insulin-like peptide receptor, and anaplastic lymphoma kinase clades of *Bombus*, *Drosophila*, and mosquitoes. The tree was rooted using the muscle-specific kinase gene from *D. melanogaster*. Node size indicates the bootstrap values (1,000 replicates). The accession numbers of amino acid sequences are labeled in front of the species names.

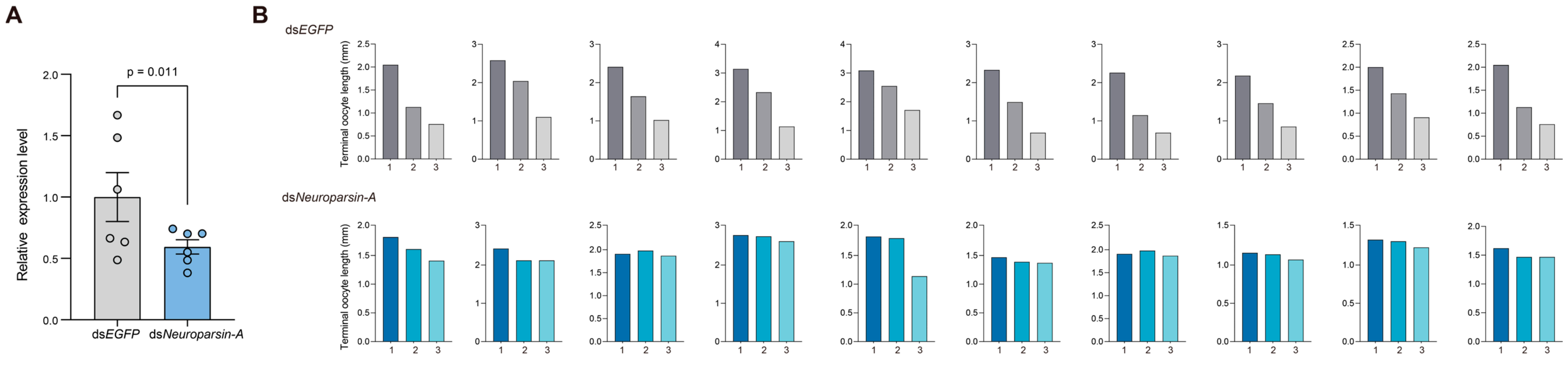

Figure S8. Differential expressions of *Neuroparsin-A* and terminal oocyte length with RNAi.

(A) Relative expressions of the *Neuroparsin-A* gene in brain of queenless groups on day 7 before and after RNAi. Statistical analysis was analyzed using the Mann–Whitney *U* test (n = 6). Each dot represents the average relative expression level of *Neuroparsin-A* gene from three workers in the same queenless group.

(B) Terminal oocyte length of workers from 10 replicating queenless groups treated with *Neuroparsin-A* or *EGFP* dsRNA.

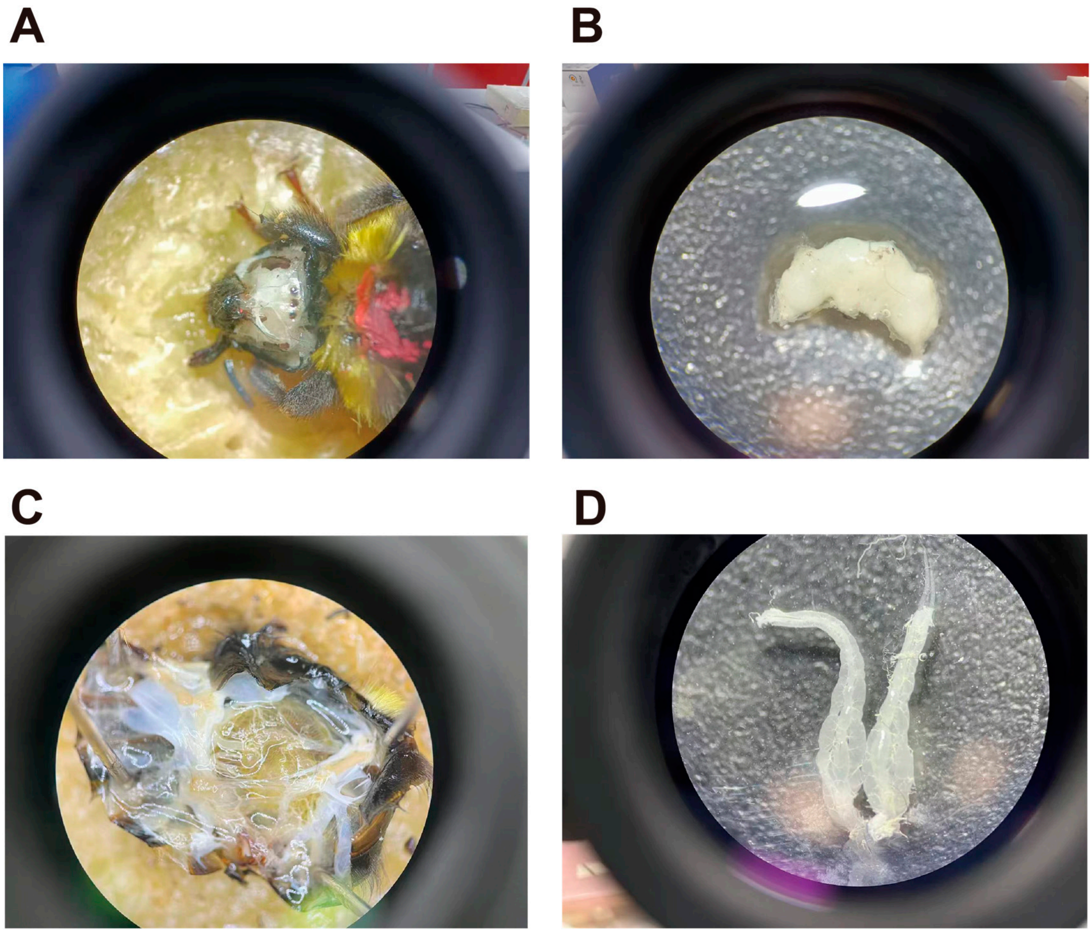

Figure S9. Anatomy of the bumblebee brain and ovary.
(A) Removal of the head cuticle. (B) Dissection and isolation of the intact brain. (C) Exposure and extraction of internal organs. (D) Dissection and preparation of ovaries.

Supplementary Videos

Video S1. Pumping behavior of the dominant bee in the queenless group.

Video S2. Buzzing behavior of the dominant bee in the queenless group.
