# Supplementary material for: The Neuropeptide Neuroparsin-A Regulates the Establishment of Dominance Hierarchy in Bumblebees
Source: Int J Mol Sci. 2025 Dec 21;27(1):91. doi: 10.3390/ijms27010091 (PMC12785732; doi:10.3390/ijms27010091)
Supplement: Supplementary file 1 [file ijms-27-00091-s001.zip › Supplementary file Table S1 Pumping behavior events recorded in different queenless groups (n = 10) during the establishment of dominance hierarchy.pdf]

Table S1. Pumping behavior events recorded in different queenless groups (n = 10) during the establishment of dominance hierarchy.

Dominance rank	Pumping behavior						
	Day 1	Day 2	Day 3	Day 4	Day 5	Day 6	Day 7
α	8.5±0.6 a	8.5±0.8 a	13.0±1.1 a	8.2±0.9 a	7.4±0.7 a	5.8±0.6 a	5.1±1.1 a
β	5.8±1.0 b	4.8±0.5 b	6.0±0.8 b	3.4±0.4 b	2.3±0.4 b	2.2±0.3 b	0.7±0.4 b
γ	4.5±0.3 b	3.3±0.5 b	2.8±0.3 c	1.4±0.3 b	1.3±0.4 b	0.7±0.3 b	0.2±0.1 b

Different letters in each column indicate significant differences determined by One-way ANOVA ($p < 0.05$).
