# Supplementary material for: The Neuropeptide Neuroparsin-A Regulates the Establishment of Dominance Hierarchy in Bumblebees
Source: Int J Mol Sci. 2025 Dec 21;27(1):91. doi: 10.3390/ijms27010091 (PMC12785732; doi:10.3390/ijms27010091)
Supplement: Supplementary file 1 [file ijms-27-00091-s001.zip › Supplementary file Table S2 Buzzing behavior events recorded in different queenless groups (n = 10) during the establishment of dominance hierarchy.pdf]

Table S2. Buzzing behavior events recorded in different queenless groups (n = 10) during the establishment of dominance hierarchy

Dominance rank	Buzzing behavior						
	**Day 1**	**Day 2**	**Day 3**	**Day 4**	**Day 5**	**Day 6**	**Day 7**
α	1.4±0.4 a	1.0±0.4 a	1.3±0.3 a	1.2±0.3 a	0.5±0.1 a	0.3±0.1 a	0.4±0.2 a
β	1.0±0.4 a	0.7±0.3 ab	0.7±0.2 a	0.1±0.1 b	0.1±0.1 b	0.3±0.2 a	0.0±0.0 b
γ	0.5±0.3 a	0.1±0.1 b	0.3±0.1 a	0.1±0.1 b	0.0±0.0 b	0.0±0.0 a	0.0±0.0 b

Different letters in each column indicate significant differences determined by One-way ANOVA ($p < 0.05$).
