# Supplementary material for: The Neuropeptide Neuroparsin-A Regulates the Establishment of Dominance Hierarchy in Bumblebees
Source: Int J Mol Sci. 2025 Dec 21;27(1):91. doi: 10.3390/ijms27010091 (PMC12785732; doi:10.3390/ijms27010091)
Supplement: Supplementary file 1 [file ijms-27-00091-s001.zip › Supplementary file Table S3 Dominance Index values calculated for individual bees (n = 10) based on behavioral encounters within the same group.pdf]

Table S3. Dominance Index values calculated for individual bees (n = 10) based on behavioral encounters within the same group.

**Dominance rank**	**Dominance Index**						
	**Day 1**	**Day 2**	**Day 3**	**Day 4**	**Day 5**	**Day 6**	**Day 7**
α	0.59±0.04 a	0.59±0.06 a	0.66±0.07 a	0.78±0.07 a	0.91±0.03 a	0.96±0.02 a	0.88±0.07 a
β	0.54±0.06 a	0.52±0.05 ab	0.43±0.10 a	0.48±0.08 b	0.43±0.08 b	0.52±0.07 b	0.54±0.08 b
γ	0.45±0.07 a	0.34±0.08 b	0.37±0.09 a	0.24±0.07 b	0.31±0.07 b	0.11±0.06 c	0.06±0.03 c

Different letters in each column indicate significant differences determined by One-way ANOVA ($p < 0.05$).
