# Supplementary material for: The Neuropeptide Neuroparsin-A Regulates the Establishment of Dominance Hierarchy in Bumblebees
Source: Int J Mol Sci. 2025 Dec 21;27(1):91. doi: 10.3390/ijms27010091 (PMC12785732; doi:10.3390/ijms27010091)
Supplement: Supplementary file 1 [file ijms-27-00091-s001.zip › Supplementary file Table S7 Primer list.pdf]

Table S7. Primer list

Purpose	Gene	Gene Symbol in NCBI	Forward	Reverse
ds_RNAi	*Neuroparsin-A*	*LOC100647295*	TAATACGACTCACTATAGGGATATCGAATGCTTGCGGGGT	TAATACGACTCACTATAGGGGATAGCAGGCCGCCTTACAG
ds_RNAi	*EGFP*	EMWEY_00056320	TAATACGACTCACTATAGGGTGAGCAAGGGCGAGGAG	TAATACGACTCACTATAGGGCGGCGGTCACGAACTCCAG
qPCR	*Neuroparsin-A*	*LOC100647295*	GCTTGCCATCGTTCTTCTGC	CATTTGTGGCAAGAGTCGCC
qPCR	*β-actin*	*LOC100646910*	TGACGCAGATTATGTTTGAA	AGCGTATAGCGAAAGTACAGC
